# Differences of Gut Microbiota in the Freshwater Blackworm (*Lumbriculus variegatus*: Oligochaeta) in Two Different Habitat Conditions

**DOI:** 10.3390/ijerph181910298

**Published:** 2021-09-29

**Authors:** Pil Soo Kim, Yeo-Rang Lee, Yong-Su Kwon, Jin-Woo Bae, Sung-Jae Lee, Young-Seuk Park

**Affiliations:** 1 Department of Biology, Kyung Hee University, Seoul 02447, Korea; philkim@khu.ac.kr (P.S.K.); lyrenter86@gmail.com (Y.-R.L.); baejw@khu.ac.kr (J.-W.B.); 2 Division of Ecological Information, National Institute of Ecology, Seocheon-gun 33657, Korea; kwonys@nie.re.kr

**Keywords:** blackworm, *Lumbriculus variegatus*, intestine microbiota, pyrosequencing, bacterial community

## Abstract

The distribution of organisms is governed by their habitat condition. We analyzed bacterial communities in the gut of the blackworm *Lumbriculus variegatus* by pyrosequencing of the extracted intestinal metagenomic DNA. Blackworms were collected from two sampling sites with differences in irradiance and riparian vegetation, where site GP7 was covered by riparian vegetation and site GP8 was exposed to sunlight. We obtained the filtered 6414 reads from three samples of each site. At GP7, 271 OTUs were identified, including 32 OTUs unique to the site, whereas at GP8, 238 OTUs were identified, including 22 unique OTUs. Among them, 18 OTUs were shared between both sites. The phylum Proteobacteria was a major component contributing 67.84% and 64.05% of sequences at sites GP7 and GP8, respectively, while each remaining phylum contributed less than 10% at both sites. The two sites differed in microbial community composition and KEGG-indicated biochemical pathways. Community indices such as species richness and Shannon diversity were higher at site GP7 than at GP8. Meanwhile, the abundance of Cyanobacteria was significantly higher at site GP8, while site GP7 showed a greater proportion of genes for membrane transport and carbohydrate metabolism, reflecting differences in food resources.

## 1. Introduction

Distribution and abundance of organisms are governed by their environmental condition [1]. In particular, the distribution of animal gut microorganisms is dependent on intestinal conditions and habitats of their hosts [2]. Host–gut microbial interactions confer benefits including nutritional support, host physiological fitness, or protection against pathogenic colonization [3,4,5]. Earthworms and microorganisms are interdependent and their interactions affect the ingestion of diets, alteration of habitat, and the biogeochemistry of ecosystems [6,7]. Microbiomes are changed during passage through the gut of earthworms [8], and affect earthworms’ vitality. The association of earthworms and microbes takes place in a process of vermicomposting, which is a biological organic waste decomposition process [6].

Many studies have been conducted on the unique intestinal microbiota in naturally or artificially fed insect larvae and adults [9,10,11]. Industrially applicable enzymes and genomic or metabolomic resources have been sourced from gut microbial species, including free-living bacteria, archaea, and eukaryotic microorganisms [12]. Reports have also identified the importance of the gut microbiome in organisms ranging from nematodes to humans [13,14,15]. In insects, gut bacterial diversity is determined by environmental conditions and host diets [16,17].

Aquatic oligochaetes are deposit feeders which consume organic materials and inhabit various environments [18,19]. As decomposers, they contribute to the restoration of organic pollutants and improvement of water quality [20,21], and are tolerant of oxygen depletion [22]. Their distribution and occurrence are influenced by factors such as turbidity and suspended solids [22,23], water mineralization [24], and the presence of organic material [23,25]. Three particular freshwater oligochaetes (*Lumbriculus variegatus*, or blackworms, *Tubifex tubifex*, and *Limnodrilus hoffmeisteri*) are used frequently in ecotoxicology and treatment of organic pollution [26]. Blackworms as bioturbating oligochaetes enhance decomposition and nitrogen cycling in urban ponds [27], and mutualistic interactions occur between oligochaete species as a result of fecal pellets of other species containing bacteria [28].

There have been no studies of gut microbial communities relating to oligochaete habitat conditions. In this study, we tested a hypothesis that community composition and diversity of the intestinal bacteria of blackworms vary depending on host specificity and host habitat. We used pyrosequencing to identify 16S rRNA in metagenomic DNA samples isolated from full intestines of blackworms collected from a stream.

## 2. Materials and Methods

### 2.1. Field Sampling

**Figure 1:**
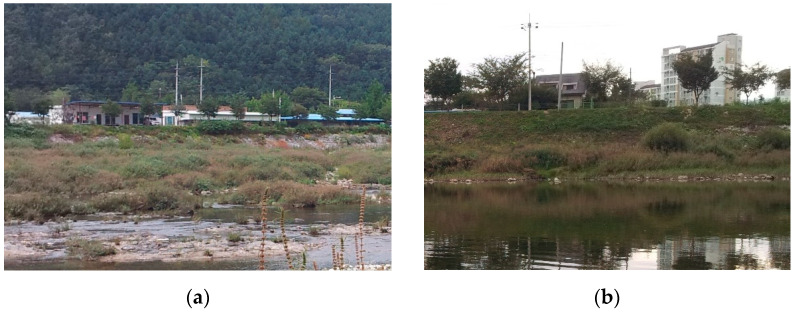
Photographs of sampling sites: GP7 (**a**) and GP8 (**b**).

Specimens of *L. variegatus* were collected at two sampling sites (GP7 and GP8) with 7 km distance from one another at the Gapyeong stream (Table 1, Figure 1) in South Korea in November 2013. The riparian vegetation at GP7 was dominated by dense stands of *Salix gracilistyla* Miq. and *Phragmites japonica* Steud., whereas GP8 had partial cover of *Phragmites japonica* and *Artemisia selengensis* growing on a sandy plain exposed to sunlight (Figure 1). Both areas were slightly polluted with 70–80 μS/cm of water electric conductivity and 12.5 mg/L of dissolved oxygen (Table 1).

A D-frame net sampler (38 cm diameter) with a mesh size of 300 µm was placed along the streambed, and the substrate was disturbed by kicking or dug by hand to dislodge blackworms living within the sediments. Collected *L. variegatus* were black, 3–4 cm long, and 1 mm in width. Three replicate samples from each site were placed in a bottle with water from the stream and chilled on ice prior to transport to the laboratory. Five individuals were randomly selected from each sample, and pooled together at each sample for gut microbial examination because the body of the specimen was small. Therefore, six pooled samples (=three samples from two sites) were used in the analyses.

### 2.2. DNA Extraction

To identify the species name of the specimens using 18S rRNA, PCR amplification was performed with purified genomic DNA, which was extracted by a PowerSoil DNA Isolation Kit (MOBIO Laboratories, Carlsbad, CA, USA). The sequence analysis was performed by Macrogen, South Korea, and the species name *Lumbriculus variegatus* was determined based on NCBI blast with a sequence showing the highest identity (97%).

Whole *L. variegatus* individuals were rinsed several times with sterile water and ethanol (70%, *v*/*v*) to exclude transient bacteria derived from the environment. Each sample was then homogenized by shaking in a sterile tube containing zirconia and glass beads of various size with 750 mL lysis buffer (500 mM NaCl, 50 mM Tris-HCl, pH 8.0, 50 mM EDTA, and 4% sodium dodecyl sulfate) for 50 s, using FastPrep-24 (MP Biomedicals, Irvine, CA, USA). Genomic DNA from the homogenized samples was extracted using phenol-chloroform precipitation and the extracted DNA was purified using an UltraClean Microbial DNA Isolation Kit (MOBIO Laboratories, Carlsbad, CA, USA) for genomic DNA purification.

### 2.3. Pyrosequencing of Bacterial 16S rRNA Genes

Bacterial compositions of blackworm guts were determined by PCR amplification of purified genomic DNA by use of Ex Taq PreMix (TaKaRa, Kyoto, Japan). PCR amplification of the 16S rRNA V1–V3 hypervariable regions was performed using the following primers, which contain an adapter sequence (A), linker sequences (TC or CA), and an eight-base sample-specific barcoded sequence (designated X): barcoded 8F (5′-A-X-TC-AGAGTTTGATCCTGGCTCAG-3′) and 518R (5′-A-X-CA-TGCTGCCTCCCGTAGGAGT-3′). Each sample was amplified in three technical replicates. PCR followed a sequence of initial denaturation at 94 °C for 3 min, followed by 30 cycles of denaturation at 94 °C for 30 s, annealing at 53 °C for 45 s, extension at 72 °C for 1 min, and a final extension step of 6 min at 72 °C. Potential contamination of buffers and primer sets was checked using DNA-free samples. PCR products were pooled with three replicates and purified using a QIAquick PCR purification kit (Qiagen, Germany). Equimolar amplicon quantities were combined and DNA quality was evaluated using the Quant-it PicoGreen dsDNA Assay Kit (Life Technologies, Carlsbad, CA, USA) on a Bioanalyzer 2100 with a DNA1000 lab chip (Agilent, Santa Clara, CA, USA). Pooled DNA samples were then amplified by emulsion PCR and 454 pyrosequencing was performed by Macrogen, South Korea, using a GS FLX Titanium system according to the manufacturer’s instructions (Roche 454 Life Sciences, Basel, Switzerland).

### 2.4. Data Analysis

We used Quantitative Insights into Microbial Ecology (QIIME; version 1.9.1) to analyze bacterial 16S rRNA sequences [29]. Raw 16S rRNA amplicon sequences from GS FLX pyrosequencing runs were filtered by quality score and length distribution. Sequences with quality score less than 25, and those shorter than 200 bp or longer than 1000 bp in length, were discarded. Post-filtered sequences were denoised using QIIME denoising algorithms (denoise_wrapper.py) [30]. Detailed read counts of the crude and filtered sequences of each sample used in this study were provided in Supplementary Table S1. These sequences were clustered into operational taxonomic units (OTUs) at 97% sequence similarity threshold using UCLUST (v5.2.236) in the QIIME pipeline [31]. The latest released QIIME-compatible version of SILVA reference database (release version 132) was used for open-reference OTU selection [32]. The ChimeraSlayer tool was used to check chimeric sequences in silico [33]. A representative sequence for each OTU was selected and aligned by Python Nearest Alignment Space Termination (PyNAST) [34]. Taxonomic assignment of the representative OTUs was carried out using the SILVA database with UCLUST. The sequence reads assigned to the chloroplast origin (based on SILVA DB) were excluded in further processes. An even-depth single rarefied OTU table (at minimum retained reads; at 829 read counts) was generated for calculations of various alpha- and beta-diversity indices and further processing. A phylogenetic tree of the normalized OTU table was constructed using the QIIME pipeline (make_phylogeny.py).

Bacterial communities were evaluated according to alpha diversity indices, including the Shannon diversity index, Faith’s phylogenetic diversity, observed OTUs and Chao1 richness. For beta diversity metrics calculation, UniFrac distances for taxonomic features were generated using the pre-calculated phylogenetic tree, and Bray–Curtis dissimilarity metrics for predicted functional features were generated using the QIIME pipeline (*beta_diversity.py*). Principal coordinates analysis (PCoA) plots were drawn using the beta-diversity metrics (UniFrac distance and Bray–Curtis dissimilarity metrics) in the QIIME pipeline (*beta_diversity_through_plots.py*). We used PICRUSt to examine the functional profiles of the bacterial community [35], where OTU tables were constructed using closed-reference selection methods against the May 2013 Greengenes database in QIIME [36]. The constructed OTU table was normalized by 16S rRNA gene copy number for correction of over- and under-estimation of abundance. The normalized dataset was compared to the Kyoto Encyclopedia of Genes and Genomes (KEGG) Orthology (KO) Dataset [37]. Each predicted functional category was presented as a KO hierarchy level.

### 2.5. Statistical Analysis

Statistical analyses were performed using GraphPad Prism (v. 8.2.1; GraphPad Software, San Diego, CA, USA). Unpaired *t*-tests were used to compare microbial composition and alpha diversity between sites. Intra-group and inter-group differences were assessed using a two-tailed Mann–Whitney *U*-test. We performed a linear discriminant analysis (LDA) effect size (LEfSe) analysis to explain differences in relative abundance of bacterial taxa by coupling standard tests with statistical significance [38] to identify differences between the two sites’ taxonomy and functional features (http://huttenhower.sph.harvard.edu/galaxy/ (accessed on 2 July 2020)). The α value used for factorial Kruskal–Wallis testing among classes was 0.05 and the thresholds for logarithmic LDA scores for discriminative features were 2.0 and 3.0 for taxonomy and functional features, respectively.

## 3. Results

### 3.1. Gut Microbiome Diversity

**Figure 2:**
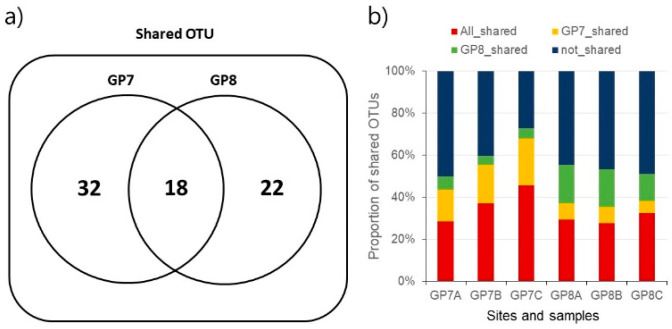
Shared core OTUs between sampling sites (**a**) and among samples (**b**).

Of 8555 total reads, 6414 reads remained after the sequence filtration against with a low quality and chloroplast origin. Samples from each site showed high fidelity with an error rate of <0.05% probability. At GP7, 271 OTUs were identified, including 32 OTUs unique to the site (Figure 2a). At GP8, 238 OTUs were identified, including 22 unique OTUs. Among them, 18 OTUs were shared between both sites. The shared OTUs were higher among samples in the same site than between sites (Figure 2b). In keeping with its greater species richness, community diversity indices were higher at GP7 than at GP8: Shannon diversity, respectively, with 6.13 and 5.86 (*t*-test, *p* < 0.05), Faith’s phylogenetic diversity with 12.28 and 10.40 (*t*-test, *p* > 0.05), and Chao1 richness with 246.89 and 207.12 (*t*-test, *p* < 0.05) (Supplementary Table S2).

**Figure 3:**
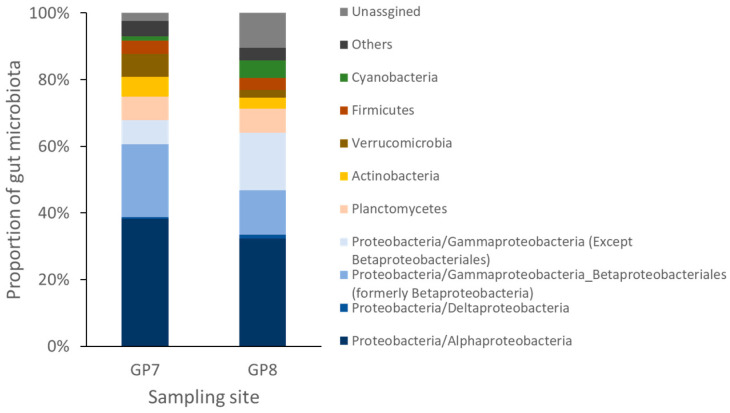
Bacterial diversity of gut microbiota 16S rRNA from blackworm *Lumbriculus variegatus* collected from two different sampling sites.

The bacterial community was dominated by the phylum Proteobacteria at both sampling sites, although its relative abundance varied (67.84% and 64.05% at GP7 and GP8, respectively; Figure 3). At GP7, Planctomycetes (7.06%) was the second most prominent taxon, followed by Verrucomicrobia (6.80%), Actinobacteria (6.02%), and Firmicutes (3.99%). At GP8, Planctomycetes (7.19%) and Cyanobacteria (5.23%) were the second most prominent taxon. Relative abundances of Actinobacteria, Verrucomicrobia, and Chloroflexi differed significantly between sites (Figure 3 and Supplementary Table S3). There were nine and six OTU samples with more or less than 95% sequence identity, respectively. *Rhodobacter* showed the highest abundance (9.90%), followed by *Achromobacter* (9.48%), *Arenimonas* (8.79%), and *Pedomicrobium* (7.03%) (Supplementary Table S4). The shared or not shared OTU numbers between two sampling sites were also compared (Supplementary Table S5).

### 3.2. Dominant Taxa Specificity

**Figure 4:**
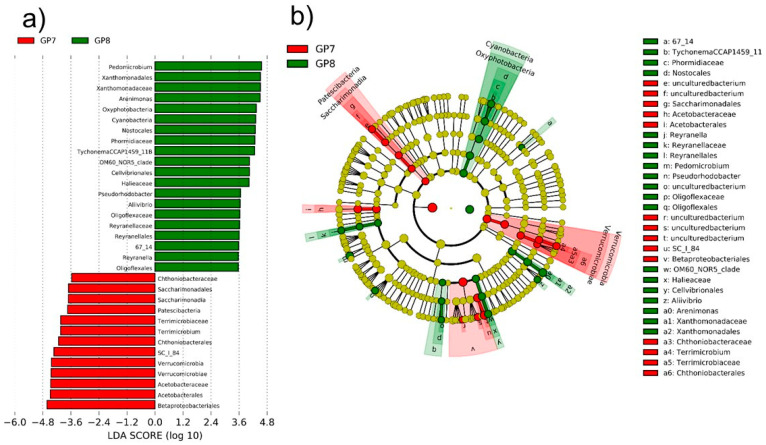
Significantly different bacterial taxa abundances between two sample sites (**a**) and phylogenetic location of significantly dominant taxa (**b**) according to linear discriminant analysis score over 2 in a LEfSe analysis.

LefSe results indicated that two sampling sites had significantly different dominant taxa (Figure 4). The dominant 13 OTUs at GP7 included 6 OTUs from the phylum *Verrucomicrobia* (2 *Chthonibacteraceae*, 2 *Terrimicrobiaceae*, and 2 *Verrucomicrobiae*), 3 OTUs from the phylum *Patescibacteria* including *Saccharimonadales*, 4 OTUs from the phylum *Proteobacteria* of *Alphaproteobacteria* including *Acetobacteraceae* and 2 *Betaproteobacteria* including SC_I_84. At GP8, 14 OTUs of the phylum *Proteobacteria* (5 *Alphaproteobacteria*, 7 *Gammaproteobacteria*, and 2 *Oligoflexaceae* OTUs), 5 OTUs from the phylum *Cyanobacteria* including *Oxyphotobacteria*, *Nostocales*, *Phormidiaceae*, and *Tychonema*, and an unclassified OTU 67_14 were dominant (Figure 4). The dominant bacteria were clustered separately in the phylogenetic tree, with the exception of OTU SC_I_84 at GP7, which was closer to the cluster of *Proteobacteria* taxa at GP8 (Figure 4). Therefore, despite the phylum *Proteobacteria* being a major component at both GP7 and GP8, the phyla *Verrucomicrobia* and *Patescibacteria*, and the phylum *Cyanobacteria* are dominant depending on site at GP7 and GP8, respectively.

**Figure 5:**
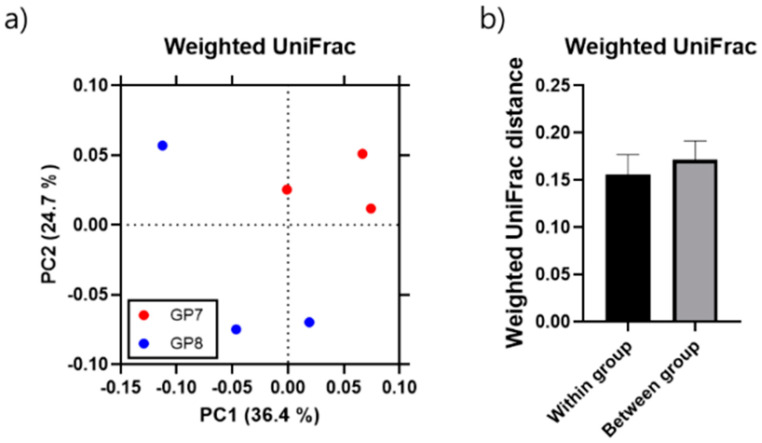
Weighted principal coordinates analysis (PCoA) with the first and second principal components (PC1 and PC2) explaining 36.4% and 24.7% of the variance, respectively (**a**); inter-(within) and intra-(between) diversity analysis between two sampling sites and among three samples from each site, respectively, and data are presented as mean ± standard deviations (**b**).

All samples from both sites showed scattering of the relative composition of bacterial OTUs on the PCoA plot (36.4% and 24.7% for PC1 and PC2, respectively), with samples clearly separated along PC1 according to site (Figure 5). Two of three samples were closely positioned at each site. The inter-diversity of the within-group was significantly lower than intra-diversity between groups at both sites (two-tailed Mann–Whitney *U*-test, *p* < 0.001; Figure 5), indicating that gut microbiome diversity has dominant taxa specificity.

### 3.3. Functional Significance of Core Microflora

**Figure 6:**
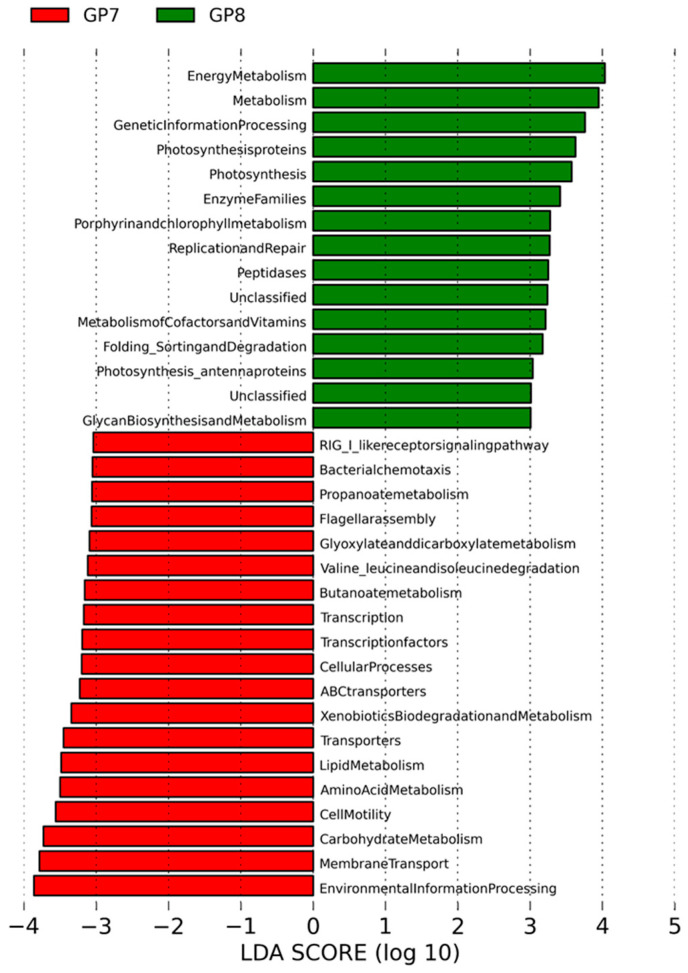
Genetic functional categories of genomic contents according to KEGG pathway analysis between two sites, with the list of categories indicating LDA scores > 3.

**Figure 7:**
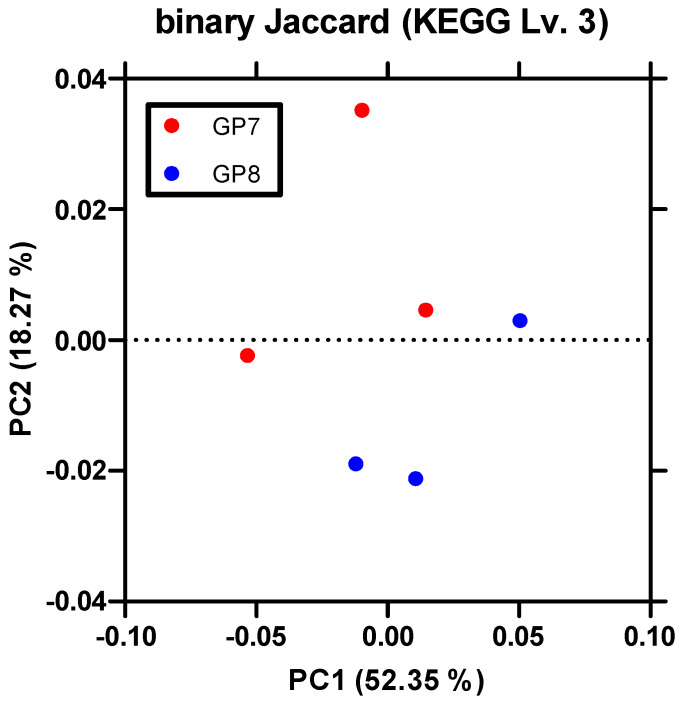
Principal coordinates analysis (PCoA) of PICRUSt_KEGG scattering according to the Binary Jaccard algorithm with PC1 and PC2 explaining 52.35% and 18.27% of the variance, respectively.

A comparison of KEGG pathways between sampling sites indicated that at GP7, significant genes for membrane transport, carbohydrate transporter, lipid and amino acid metabolism, transcription, and cellular motility were remarkably revealed. At GP8, genes related to photosynthesis, energy metabolism, replication, and repair were identified (Figure 6). Sampling sites were scattered according to these differences in KEGG pathways in PCoA (Figure 7).

## 4. Discussion

We compared gut bacteria associated with the blackworm *L. variegatus* between two sampling sites with tiny environmental differences in nutrients and solar irradiance in a freshwater stream.

### 4.1. Gut Bacterial Community

We detected a core microbiota from all blackworm samples consisting of 6414 reads, with the phylum *Proteobacteria* dominating with 67.84% for GP7 and 64.05% for GP8 (Figure 2). Baquiran et al. [39] showed that *Proteobacteria* and *Sphingobacteria* were the predominant taxa in the nematode species *Acrobeloides maximus* and *Caenorhabditis elegans*, which comprised ca. 30% and 60%, and 60% and 20%, respectively. Dubilier et al. [40] reported endosymbionts of marine oligochaete *Inanidrilus leukodermatust* and *Proteobacteria*. Core microbiota presents an intrinsic symbiont from different environmental habitats. In this study, the top 15 OTUs mostly contain *Proteobacteria* such as core microbiota from 18 all-shared organisms (Supplementary Table S4). The *Proteobacteria* generally dominate in natural ecosystems including soil (36.5%), leaves (62%), air (77.9%), seawater (57.9%), and freshwater (61.3%) [41]. Therefore, our results support that the gut microbiome of freshwater oligochaetes mostly has the phylum *Proteobacteria* without *Sphingobacteria* that dominate other nematode species’ specificity.

### 4.2. Differences in Gut Microbiome in Distinct Habitat Conditions

Animal gut microbiota can be determined by intestinal shape, food resources, and environmental conditions [14,16,42]. These factors could explain the variation in host-specific gut microbiota in blackworms. Our samples were collected at two different sites with different environmental conditions, specifically nutrients and solar irradiation. The relative abundance of *Cyanobacteria* 16S rRNA gene sequences was relatively higher at GP8 than at GP7 (*p* = 0.1), with KEGG pathway findings indicating genes related to photosynthesis and energy metabolism. By contrast, GP7 pathways were related to membrane transport and carbohydrate metabolism. Although both sampling sites have slightly different abiotic environmental factors, especially electric conductivity (Table 1), this difference in composition of these microbiomes may be due to exposure to sunlight at relatively low vegetation GP8 in the riparian area. Blackworms at this location would have greater opportunity to consume photosynthesizing organisms. Therefore, this difference in habitat can be explained by factors such as sunlight, canopy coverage, riparian vegetation, and food resources, which have an important impact on gut microbial communities.

Balykin [43] isolated symbiotic bacteria from the genus *Bacillus* in the gut of tubificids, and demonstrated a relationship between the number of microorganisms in the substrate and the gut. Symbiotic interactions play an important role in recycling organic materials, contributing to biochemical and physiological processes of oligochaetes [43,44]. In this study, we did not determine which bacterial species constitute symbionts and which constitute prey.

### 4.3. Limitation and Further Studies

Because the phyla *Proteobacteria*, *Actinobacteria*, and *Firmicutes* are common in freshwater, we have not been able to distinguish the microbiome inhabiting the intestine from the environmental bacteria [41,45]. The environmental microbiota as well as food resources influence the intestinal microbiota [45,46,47]. Nevertheless, in this study, the two sampling sites had different environmental conditions and the formation of gut microbiome in blackworms might be affected. We did not consider the phenotypic differences of blackworms between the two sampling sites. Differences in phenotype may be related to differences in blackworm population genetics that potentially affect their gut microbiota. Therefore, further studies are required (1) to evaluate the relationship between symbiotic microbiota in the gut, other intestinal microbiota and environmental microbiota of more samples from various habitat conditions, (2) to define the specific roles of bacterial species identified in the gut of blackworms using growth phase-dependent monitoring and cross-transplantation between sampling sites, and (3) to examine the effects of phenotype related to genetics in different populations in the gut microbiome of oligochaetes.

## 5. Conclusions

We collected blackworms from two sampling sites with different habitat conditions in irradiance and riparian vegetation, and analyzed bacterial communities in the gut of the blackworm. The phylum *Proteobacteria* was the dominant taxa at both sampling sites. However, the two sampling sites had different microbial community compositions and KEGG-indicated biochemical pathways. The abundance of taxa with chloroplasts was significantly higher at a site exposed to sunlight, while a greater proportion of genes for membrane transport and carbohydrate metabolism was observed at a site covered by riparian vegetation. Therefore, our results present a possibility that habitat conditions modulated the composition of the gut microbiota and their biochemical properties in the freshwater blackworms.

## Figures and Tables

**Table 1:** Environmental characteristics (mean ± standard error) at sampling sites.

Environmental Variable	Sampling Site	
	GP7	GP8
Location (latitude, longitude)	37°52′27″ N, 127°31′34″ E	37°50′03″ N, 127°30′57″ E
Water velocity (m/s)	0.3 ± 0.1	0.2 ± 0.0
Water depth (cm)	46.9 ± 9.3	48.2 ± 10.9
Water width (m)	47.8 ± 2.5	77.5 ± 19.3
Turbidity (NTU)	1.3 ± 0.4	1.6 ± 0.3
Electric conductivity (μS/cm)	69.8 ± 3.2	79.7 ± 4.5
pH	7.5 ± 0.1	7.7 ± 0.2
Dissolved oxygen (mg/L)	12.6 ± 0.5	12.4 ± 0.5

## Data Availability

The 16S rRNA gene sequences of blackworm gut microbiota were submitted to the DDBJ Sequence Read Archive (DRA) of DDBJ (DNA Data Bank of Japan) under the accession number PRJDB8800.

## Supplementary Materials

The following are available online at https://www.mdpi.com/article/10.3390/ijerph181910298/s1, Table S1: The number of sequences of the crude and filtered read counts in samples that used in this study. Table S2: The values of alpha diversity metrics comparison between sampling sites GP7 and GP8. Table S3: The relative abundance (%) of phyla in 6 samples from sampling sites GP7 (GP7A-C) and GP8 (GP8A-C). Table S4: The top 15 OTUs in 6 samples from sampling sites GP7 (GP7A-C) and GP8 (GP8A-C). Table S5: Comparison of shared or not-shared OTU numbers between sampling sites GP7 and GP8.
