# Supplementary material for: Differences of Gut Microbiota in the Freshwater Blackworm (Lumbriculus variegatus: Oligochaeta) in Two Different Habitat Conditions
Source: Int J Environ Res Public Health. 2021 Sep 29;18(19):10298. doi: 10.3390/ijerph181910298 (PMC8508024; doi:10.3390/ijerph181910298)
Supplement: Supplementary file 1 [file ijerph-18-10298-s001.zip › ijerph-1354904-supplementary.pdf]

Differences of gut microbiota in the freshwater blackworm (*Lumbriculus variegatus*: Oligochaeta) at two different habitat conditions

Pil Soo Kim, Yeo-Rang Lee, Yong Su Kwon, Jin-Woo Bae, Sung-Jae Lee and Young-Seuk Park

Supplementary information:
- Table S1-S5

Table S1. The number of sequences of the crude and filtered read counts in samples that used in this study.

Samples	Crude read counts (No. of non-filtered seq.)	Filtered read counts (No. of quality-filter passed and singleton excluded seq.)
GP7A	2018	1764
GP7B	1020	899
GP7C	1119	1023
GP8A	906	827
GP8B	1977	1840
GP8C	1515	1432

Table S2. The values of alpha diversity metrics comparison between sampling sites GP7 and GP8. The asterisks indicate significant differences according to two-tailed unpaired *t*-test between sampling sites GP7 and GP8 ($p < 0.05$).

**Alpha diversity metrics**	**GP7**	**GP8**
Shannon diversity *	6.13	5.86
Faith's phylogenetic diversity *	12.28	10.40
Chao1 richness	246.89	207.12
Observed OTUs *	149.33	129.67

Table S3. The relative abundance (%) of phyla in 6 samples from sampling sites GP7 (GP7A-C) and GP8 (GP8A-C).

**Phylum**	**GP7**			**GP8**			
	**GP7A**	**GP7B**	**GP7C**	**GP8A**	**GP8B**	**GP8C**	***p-value***
**Proteobacteria**	63.14	73.14	67.25	60.98	63.14	68.04	0.177
Alphaproteobacteria	*28.63*	*43.33*	*42.75*	*29.61*	*34.51*	*32.94*	0.173
*Betaproteobacteriales (formerly Betaproteo bacteria)**	*24.31*	*23.73*	*17.45*	*13.14*	*11.57*	*15.29*	0.021
*Gammaproteobacteria (Except Betaproteob acteriales)***	*9.22*	*5.49*	*7.06*	*17.65*	*16.27*	*17.84*	0.002
Deltaproteobacteria	*0.98*	*0.59*	*0.00*	*0.59*	*0.78*	*1.96*	0.163
**Planctomycetes**	9.61	3.53	8.04	5.49	9.41	6.67	0.478
**Actinobacteria**	6.67	7.65	3.73	3.73	4.12	2.35	0.072
**Verrucomicrobia***	3.92	6.86	9.61	4.71	1.37	0.78	0.049
**Firmicutes**	3.92	3.14	4.90	1.57	6.27	2.94	0.406
**Cyanobacteria**	2.75	1.18	0.20	3.53	1.57	10.59	0.146
**Fusobacteria**	0.98	0.39	0.20	0.59	5.49	0.78	0.193
**Patescibacteria**	3.33	0.39	0.98	0.39	0.20	0.39	0.150
**Chloroflexi***	0.78	0.59	0.78	0.39	0.39	0.59	0.024
**Tenericutes**	0.59	0.59	0.59	0.00	0.39	0.59	0.135
**WPS-2**	0.39	0.78	0.00	0.00	0.00	0.00	0.113
**Bacteroidetes**	0.39	0.00	0.39	0.00	0.20	0.20	0.219
**Dependentiae**	0.20	0.20	0.20	0.00	0.20	0.00	0.092
**Acidobacteria**	0.39	0.00	0.00	0.00	0.00	0.00	0.211
**Gemmatimonadetes**	0.00	0.00	0.00	0.00	0.39	0.00	0.211
**Epsilonbacteraeota**	0.00	0.00	0.00	0.00	0.20	0.00	0.211
**Rokubacteria**	0.00	0.00	0.00	0.00	0.20	0.00	0.211
Uncl. Bacteria	0.00	0.00	0.39	0.00	0.00	0.00	0.211
Unassgined	2.94	1.57	2.75	18.63	6.47	6.08	0.096

The asterisks indicate significant differences according to one-tailed unpaired t-test between sampling sites GP7 and GP8.
* ($p < 0.05$); ** ($p < 0.01$); *** ($p < 0.001$)

Table S4. The top 15 OTUs in 6 samples from sampling sites GP7 (GP7A-C) and GP8 (GP8A-C).

#OTU ID	GP7			GP8			Total Abundance (%)	Phylum	Class	Order	Family	Genus*
	GP7A	GP7B	GP7C	GP8A	GP8B	GP8C						
FTOM01000018.227.1697	6.86	8.04	33.53	2.94	2.16	5.88	9.90	Proteobacteria	*Alphaproteobacteria*	*Rhodobacterales*	*Rhodobacteraceae*	*Rhodobacter*
EF555461.1.1516	17.06	10.39	1.96	1.57	14.90	10.98	9.48	Proteobacteria	*Gammaproteobacteria*	*Betaproteobacteriales*	*Burkholderiaceae*	*Achromobacter*
GQ396821.1.1509	13.14	12.16	13.14	2.35	7.65	6.47	9.15	Proteobacteria	*Gammaproteobacteria*	*Betaproteobacteriales*	*SC-I-84*	*uncultured bacterium*
HQ178940.1.1470	9.22	3.14	5.69	8.82	12.16	13.73	8.79	Proteobacteria	*Gammaproteobacteria*	*Xanthomonadales*	*Xanthomonadaceae*	*Arenimonas*
denovo.CleanUp.ReferenceOTU306	0.00	0.00	0.00	0.20	41.96	0.00	7.03	Proteobacteria	*Alphaproteobacteria*	*Rhizobiales*	*Hyphomicrobiaceae*	*Pedomicrobium*
denovo.CleanUp.ReferenceOTU11	14.71	0.00	2.35	1.37	0.39	22.55	6.90	Proteobacteria	*Alphaproteobacteria*	*Rhizobiales*	*Beijerinckiaceae*	*alphaI cluster*
FJ592534.1.1396	16.47	1.76	0.00	4.31	0.00	8.04	5.10	Proteobacteria	*Gammaproteobacteria*	*Betaproteobacteriales*	*Rhodocyclaceae*	*Candidatus Accumulibacter*
DQ303296.1.1480	8.82	2.55	2.75	0.98	6.47	2.16	3.95	Proteobacteria	*Alphaproteobacteria*	*Rhizobiales*	*Xanthobacteraceae*	*Bradyrhizobium*
denovo.CleanUp.ReferenceOTU582	0.00	22.75	0.00	0.00	0.00	0.00	3.79	Proteobacteria	*Alphaproteobacteria*	*Acetobacterales*	*Acetobacteraceae*	
denovo.CleanUp.ReferenceOTU238	3.53	6.67	9.41	0.39	0.78	1.96	3.79	Proteobacteria	*Alphaproteobacteria*	*Rhodobacterales*	*Rhodobacteraceae*	
HQ114181.1.1456	2.16	1.18	1.76	3.53	9.61	3.92	3.69	Proteobacteria	*Gammaproteobacteria*	*Cellvibrionales*	*Halieaceae*	*OM60(NOR5) clade*
KC749107.1.1486	5.29	4.51	1.96	1.96	2.75	2.94	3.24	Proteobacteria	*Alphaproteobacteria*	*Rhizobiales*	*Rhizobiales Incertae Sedis*	*uncultured*
FPLK01000581.11.1482	6.47	2.94	3.53	1.18	4.12	0.59	3.14	Proteobacteria	*Alphaproteobacteria*	*Rhizobiales*	*Rhizobiales Incertae Sedis*	*uncultured*
EF438215.1.1399	0.00	0.00	0.00	1.76	0.00	15.29	2.84	Cyanobacteria	*Oxyphotobacteria*	*Nostocales*	*Phormidiaceae*	*Tychonema CCAP 1459-11B*
HG970980.1.1567	0.78	0.59	0.00	0.20	14.90	0.59	2.84	Fusobacteria	*Fusobacteriia*	*Fusobacteriales*	*Fusobacteriaceae*	*Fusobacterium*

Table S5. Comparison of shared or not-shared OTU numbers between sampling sites GP7 and GP8.

No of OTUs	GP7A	GP7B	GP7C	GP8A	GP8B	GP8C
**All_shared**	18	18	18	18	18	18
**GP7_shared**	32	32	32	15	19	11
**GP8_shared**	10	9	8	22	22	22
**not_shared**	119	75	77	68	84	72
